# The Fuzzy planar cell polarity protein (FUZ), necessary for primary cilium formation, is essential for pituitary development

**DOI:** 10.1111/joa.13961

**Published:** 2023-10-04

**Authors:** Emily J. Lodge, William B. Barrell, Karen J. Liu, Cynthia L. Andoniadou

**Affiliations:** ^1^ Centre for Craniofacial and Regenerative Biology King's College London London UK; ^2^ Department of Medicine III University Hospital Carl Gustav Carus, Technische Universität Dresden Dresden Germany

**Keywords:** FUZ, pituitary development, primary cilium, SHH signalling

## Abstract

The primary cilium is an essential organelle that is important for normal cell signalling during development and homeostasis but its role in pituitary development has not been reported. The primary cilium facilitates signal transduction for multiple pathways, the best‐characterised being the SHH pathway, which is known to be necessary for correct pituitary gland development. FUZ is a planar cell polarity (PCP) effector that is essential for normal ciliogenesis, where the primary cilia of *Fuz* ^*−/−*^mutant*s* are shorter or non‐functional. FUZ is part of a group of proteins required for recruiting retrograde intraflagellar transport proteins to the base of the organelle. Previous work has reported ciliopathy phenotypes in *Fuz* ^*−/−*^ homozygous null mouse mutants, including neural tube defects, craniofacial abnormalities, and polydactyly, alongside PCP defects including kinked/curly tails and heart defects. Interestingly, the pituitary gland was reported to be missing in *Fuz* ^*−/−*^ mutants at 14.5 dpc but the mechanisms underlying this phenotype were not investigated. Here, we have analysed the pituitary development of *Fuz* ^*−/−*^ mutants. Histological analyses reveal that Rathke's pouch (RP) is initially induced normally but is not specified and fails to express LHX3, resulting in hypoplasia and apoptosis. Characterisation of SHH signalling reveals reduced pathway activation in *Fuz* ^*−/−*^ mutant relative to control embryos, leading to deficient specification of anterior pituitary fate. Analyses of the key developmental signals FGF8 and BMP4, which are influenced by SHH, reveal abnormal patterning in the ventral diencephalon, contributing further to abnormal RP development. Taken together, our analyses suggest that primary cilia are required for normal pituitary specification through SHH signalling.

## Graphical abstract

This manuscript shows that the primary cilium is an essential organelle for pituitary gland development and that endocrine disturbance of the pituitary gland can be a manifestation of ciliopathies.

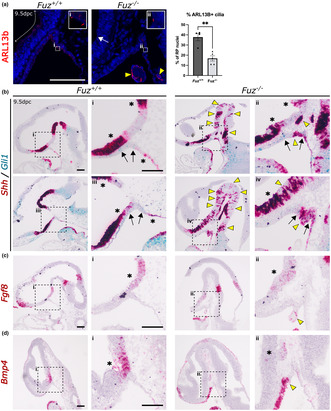

## 1 INTRODUCTION

The pituitary gland is a primary endocrine organ that produces and secretes hormones controlling vital physiological processes including metabolism, growth, fertility and the stress response. Hormone‐producing endocrine cells reside in the highly vascularised anterior pituitary, which receives signals from the hypothalamus, controlling hormone release. Multiple signalling pathways have been implicated in pituitary embryonic development, however, the requirement of a major cellular signalling hub, the primary cilium, is not well understood.

The anterior pituitary is specified during embryonic development from the oral epithelium (OE), induced by signals from surrounding tissues, primarily *Fgf8/10* and *Bmp4* from the overlying ventral diencephalon (VD) and *Shh* from both the anterior hypothalamus (AH) and pharyngeal endoderm (PE) (for review see (Willis et al., 2022)). These signals promote invagination of the oral epithelium at around 9.0 dpc forming Rathke's pouch (RP), the anterior pituitary primordium; promote its proliferation and acquisition of anterior pituitary fate by induction of transcription factors LHX3 and LHX4. At 11.5 dpc, a region of the VD evaginates towards the developing RP, forming the infundibulum, which is the primordium of the future posterior pituitary lobe. FGF/BMP and SHH signals are antagonistic, and the border between them in neural tissue specifies the rostral border of RP in the underlying oral epithelium. While *Shh* is not expressed in RP during development, its downstream target *Gli1* is highly expressed, confirming pathway activity (Carreno et al., 2017).

Signalling via the SHH pathway necessitates primary cilia as the sites where core components localise, including receptor PTCH1, co‐receptor SMO and GLI proteins that act as SHH signalling effectors and are also transcriptional targets. Without a primary cilium, SHH pathway components are localised throughout the cell, inhibiting response to the ligand, and resulting in abnormal development of multiple tissues. In mice, a reduction in SHH signalling causes a hypoplastic pituitary gland (Treier et al., 2001), whereas loss of SHH in the anterior hypothalamus results in complete arrest of RP development, no expression of *Lhx3/Lhx4*, and a loss of tissue by 12.5 dpc (Carreno et al., 2017). Furthermore, while inhibition of *Shh* is required in the ventral diencephalon for infundibulum development (Trowe et al., 2013), increasing SHH signalling in RP, results in a larger pituitary (Carreno et al., 2017).

Mutations in *SHH* are the main cause of holoprosencephaly (HPE), a failure of the embryonic forebrain to separate into left and right hemispheres, and result in multiple midline defects including hypotelorism, cleft lip and palate, and hypopituitarism (Malta et al., 2023). Clinical presentation can be highly variable within HPE, even with same variant mutations in families (Malta et al., 2023). Mutations in the SHH effector *GLI2*, have been identified in patients with HPE with abnormal pituitary function (Kelberman & Dattani, 2006) and with congenital hypopituitarism (Gregory et al., 2015). Variants of *TBC1D32*, encoding a protein implicated in SHH signalling and ciliary function, have been reported to result in hypoplastic or absent pituitary gland in humans, alongside complex ciliopathy phenotypes (Harris et al., 2023; Hietamäki et al., 2020). Furthermore, mutations in *SHH* are also associated with septo‐optic dysplasia (SOD), characterised by eye defects, midline brain abnormalities, pituitary gland anomalies and optic nerve hypoplasia (Zhao et al., 2012).

Aside from the SHH pathway the primary cilium is a major signalling site. Study of primary and motile cilia has demonstrated defects in these cellular components lead to a wide range of anomalies throughout the body, collectively known as ciliopathies (see review Focșa et al. (2021)). These include neural tube, sensory organ, respiratory, heart, liver, kidney, skeletal and reproductive system defects. Mutations in proteins of the cilia and the cilia basal body fall under this classification, however, while there are individual reports of ciliopathies with associated pituitary dysfunction, such defects are not usually included in ciliopathic phenotypes.

FUZ, is a CPLANE complex protein required for ciliogenesis, linked to vesicle trafficking and secretion and the Planar Cell Polarity (PCP) pathway and is usually located at the basal body of the cilia. In cells lacking FUZ, the cilium is shorter and is gradually lost over time (Brooks & Wallingford, 2012; Gray et al., 2009; Heydeck et al., 2009), due to requirements for retrograde transport, returning cargo from the tip (Brooks & Wallingford, 2012).

Mutations in FUZ seen in humans are associated with severe neural tube defects and perinatal lethal short‐rib polydactyly syndrome (Seo et al., 2011; Zhang et al., 2018), as well as craniosynostosis (Barrell et al., 2022). Murine *Fuz* ^*−/−*^ mutants have a range of ciliopathic and PCP phenotypes, including neural tube defects, kinked or curly tails, exencephaly, anophthalmia, brachygnathia, craniosynostosis, polydactyly and heart defects (Gray et al., 2009; Heydeck et al., 2009; Tabler et al., 2013, 2016, 2017). Furthermore, an absence of pituitary tissue was reported at 14.5 dpc (Zhang et al., 2011), however, the mechanisms underlying this phenotype were not investigated and not attributed as either a ciliopathy or PCP defect.

We describe here that *Fuz* ^*−/−*^ mutant mice undergo initial RP development, however, due to abnormal SHH, FGF8 and BMP4 signalling, the tissue does not undergo pituitary specification, becomes hypoplastic and is lost via apoptosis. Our data uncover a requirement for normal cilia in pituitary development, such that in *Fuz* ^*−/−*^ mutants reduced numbers of primary cilia in neural tissue and RP result in alterations in hypothalamic signalling and activation of the SHH pathway. This implicates hypothalamic–pituitary defects as primary endocrine defects in ciliopathies.

## 2 RESULTS

### 2.1 FUZ is expressed in the developing pituitary and is required for normal development

**FIGURE 1:**
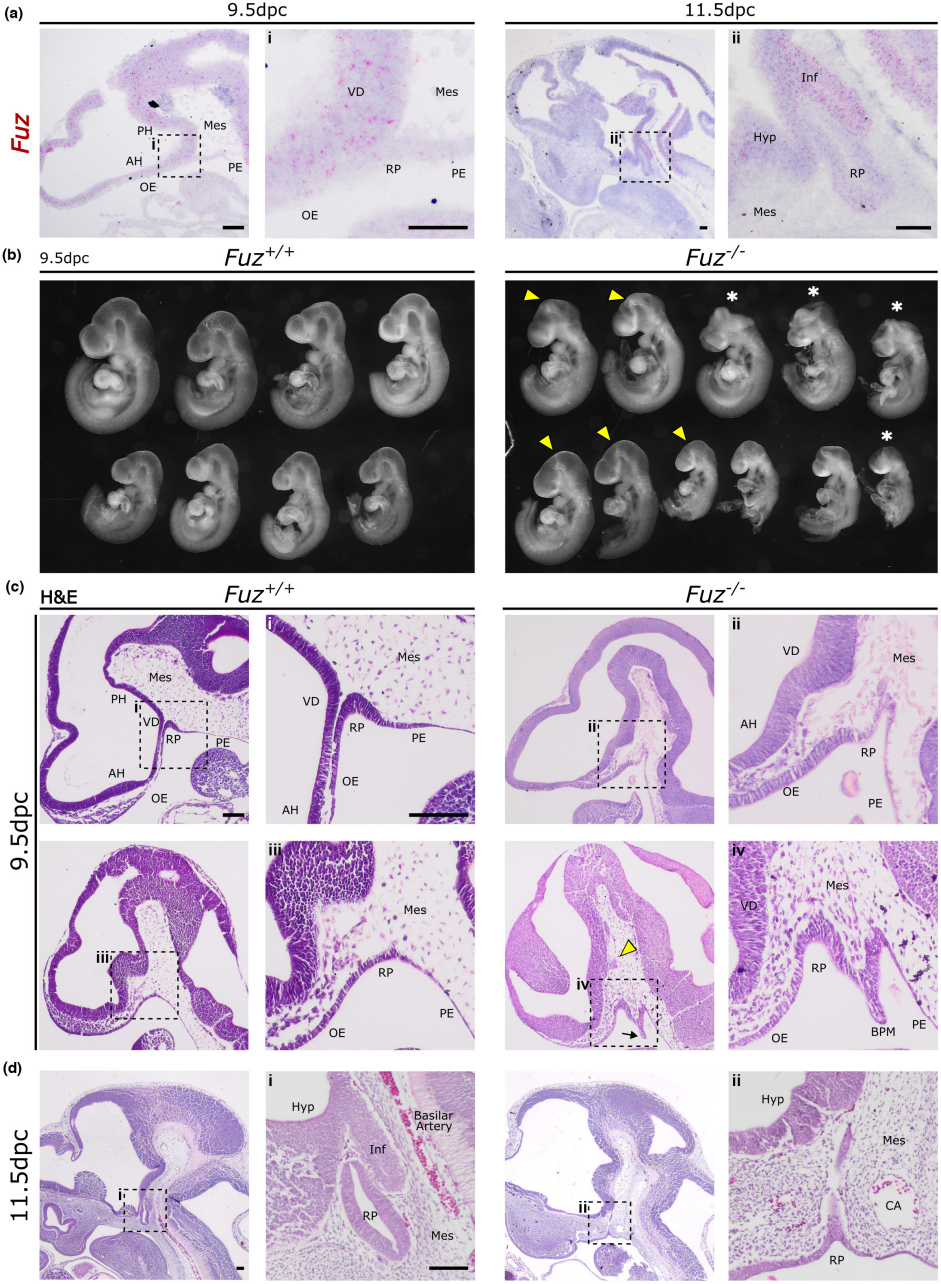
*Fuz* is expressed in the embryonic pituitary and is required for normal development. (a) mRNA in situ hybridisation for using probes for *Fuz* at stages 9.5 dpc and 11.5 dpc in WT mouse, counterstained with hematoxylin. At 9.5 dpc, *Fuz* is expressed throughout neural tissues and invaginating Rathke's pouch (RP). At 11.5 dpc, *Fuz* is expressed at higher levels in the infundibulum, than in RP, which shows a gradient of expression from dorsal to ventral. (b) Whole‐mount images of dissected 9.5 dpc *Fuz* ^*+/+*^ and *Fuz* ^*−/−*^ mutant embryos. Yellow arrowheads indicate tight mesencephalic flexure; asterisks denote embryos with neural tube closure defects. Hematoxylin and eosin staining of midline sagittal sections from WT (*Fuz* ^*+/+*^) and *Fuz* ^*−/−*^ showing low‐magnification views of the head, and higher magnification of the invaginating RP, shown by dotted selection at 9.5 dpc (c) and 11.5 dpc (d). At 9.5 dpc mutant shows abnormal protrusion of neural tissue (yellow arrowhead) and remnants of buccopharyngeal membrane (BPM, arrow). In 11.5 dpc samples, WT shows definitive pouch and infundibulum, while mutant is hypoplastic and still connected to OE. AH, anterior hypothalamus; BPM, buccopharyngeal membrane; CA, carotid artery; Hyp, hypothalamus; Inf, infundibulum; Mes, mesenchyme; OE, oral epithelium; PE, pharyngeal endoderm; PH, posterior hypothalamus; RP, Rathke's pouch; VD, ventral diencephalon. All scale bars 100 μm.

We first sought to confirm expression of *Fuz* within the developing murine pituitary by RNAscope mRNA in situ hybridisation. At 9.5 dpc, when correct signalling is key for the initial organ specification, *Fuz* mRNA transcripts are present in the anterior hypothalamus and ventral diencephalon, as well as throughout the developing neural tissue (Figure 1a). We observe lower expression levels within Rathke's pouch (RP), oral epithelium (OE) and pharyngeal endoderm (PE). By 11.5 dpc, following closure of RP (definitive RP) and when the anterior pituitary primordium begins to expand, expression of *Fuz* is highest in the infundibulum, the future posterior lobe and the hypothalamus. There are lower levels of *Fuz* mRNA transcripts within RP, displaying dorsoventral gradient, with lower expression observed in less differentiated cells of the presumptive anterior pituitary. RNAscope mRNA in situ hybridisation for *Fuz* on *Fuz* ^*−/−*^ homozygous mutant mouse sections at these ages confirms complete loss of expression as no transcripts are detected (Figure S1A).

Intercrosses of *Fuz* ^*+/−*^ x *Fuz* ^*+/−*^ heterozygous mice were collected at three stages: 9.5 dpc, 11.5 dpc and 13.5 dpc. Whole‐mount images of 9.5 dpc embryos, shown in Figure 1b, demonstrate variable phenotype expressivity, where exencephaly is observed in *Fuz* ^*−/−*^ mutants (six of 19 embryos, Figure 1b asterisks) as well as developmental delay (six of 19 embryos). These phenotypes were previously described in this mutant line (Gray et al., 2009). The mesencephalic flexure between the forebrain and midbrain in *Fuz* ^*−/−*^ embryos also appears more acute (Figure 1b yellow arrowheads) as previously reported in a different FUZ mouse line (Heydeck et al., 2009).

Haematoxylin and eosin (H&E) staining of midline sagittal sections of wild type embryos (*Fuz* ^*+/+*^) shows the close contact between the invaginating RP and the overlying VD, however, in *Fuz* ^*−/−*^ embryos, the invaginating OE does not make contact and is separated from the VD by mesenchymal cells (Figure 1c). Furthermore, there were remnants of the buccopharyngeal membrane (Figure 1c, BPM, black arrow) in multiple mutant samples (*n* = 4/10) and an abnormal protrusion of neural tissue was also present in several samples (Figure 1c, yellow arrowhead) (*n* = 2/10).

H&E staining at 11.5 dpc shows the definitive RP in WT samples, with invagination of the infundibulum from VD tissue (Figure 1d). However, in *Fuz* ^*−/−*^ samples, RP is hypoplastic, still connected to the oral epithelium, and no infundibular tissue was observed. In exencephalic samples, no RP tissue was located (Figure S1C). By 13.5 dpc no pituitary tissue could be identified in mutants (*n* = 3, not shown).

### 2.2 Loss of FUZ affects *Fgf8*, *Bmp4* and *shh* expression

**FIGURE 2:**
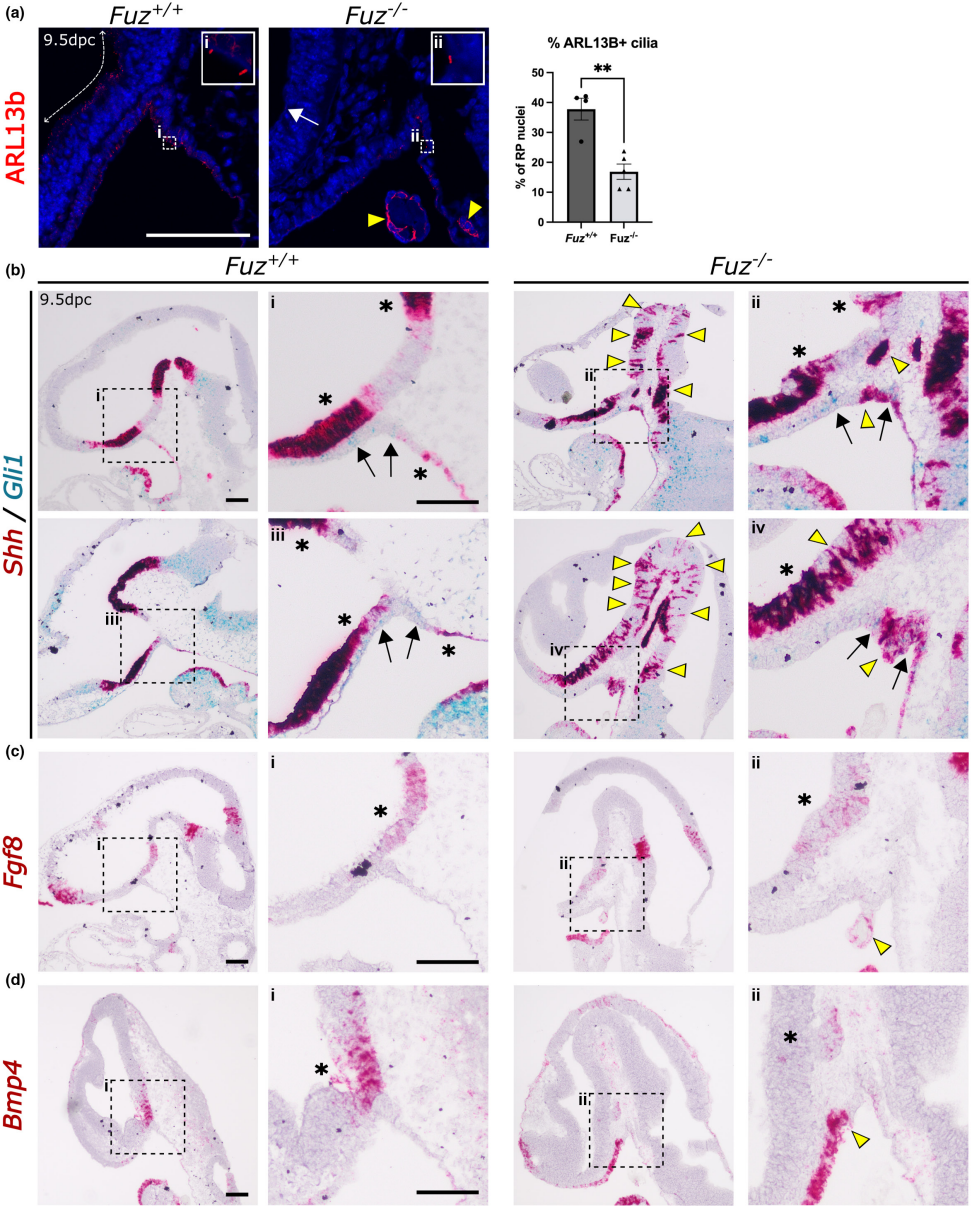
Loss of *Fuz* affects number of cilia and hypothalamic SHH signalling. (a) Immunofluorescence staining of 9.5 dpc sagittal sections for ARL13b, a cilia marker, in WT (*Fuz* ^*+/+*^) and *Fuz* ^*−/−*^. Dotted arrows mark ciliated neural tissue of the ventral diencephalon in WT, white arrow in *Fuz* ^*−/−*^ marks an individual cilium on neural tissue, yellow arrowheads indicate cytoplasmic staining in regressing BPM tissue. Dashed box indicates region for Inset zoom, which shows individual cilia in RP tissue. Graph depicts the number of cilia expressed as percent of total counted nuclei in RP tissue, there are significantly fewer ciliated cells in *Fuz* ^*−/−*^ (16.9%) than WT (37.8%), Student's t‐test, *p* = 0.0019 (**). WT *n* = 4 and *Fuz* ^*−/−*^ *n* = 5. mRNA in situ hybridisation of 9.5 dpc WT (*Fuz* ^*+/+*^) and *Fuz* ^*−/−*^ embryos, using probes (b) co‐detecting *Shh* (red) and *Gli1* (blue); single detection of (c) *Fgf8*, (d) *Bmp4;* counterstained with hematoxylin. Lower magnification image showing head, and higher magnification image showing RP, depicted by dotted box. Asterisks indicate *Shh* expression in AH, PH and PE (a); *Fgf8* and *Bmp4* expression domains in the ventral diencephalon (c, d); arrows in (a) indicate *Gli1* expression in RP. Yellow arrowheads identify ectopic expression of *Fgf8* and *Bmp4* within OE and BPM and ectopic *Shh* expression in RP and stripes of expression in neural tissue of mutants. All scale bars 100 μm.

**FIGURE 3:**
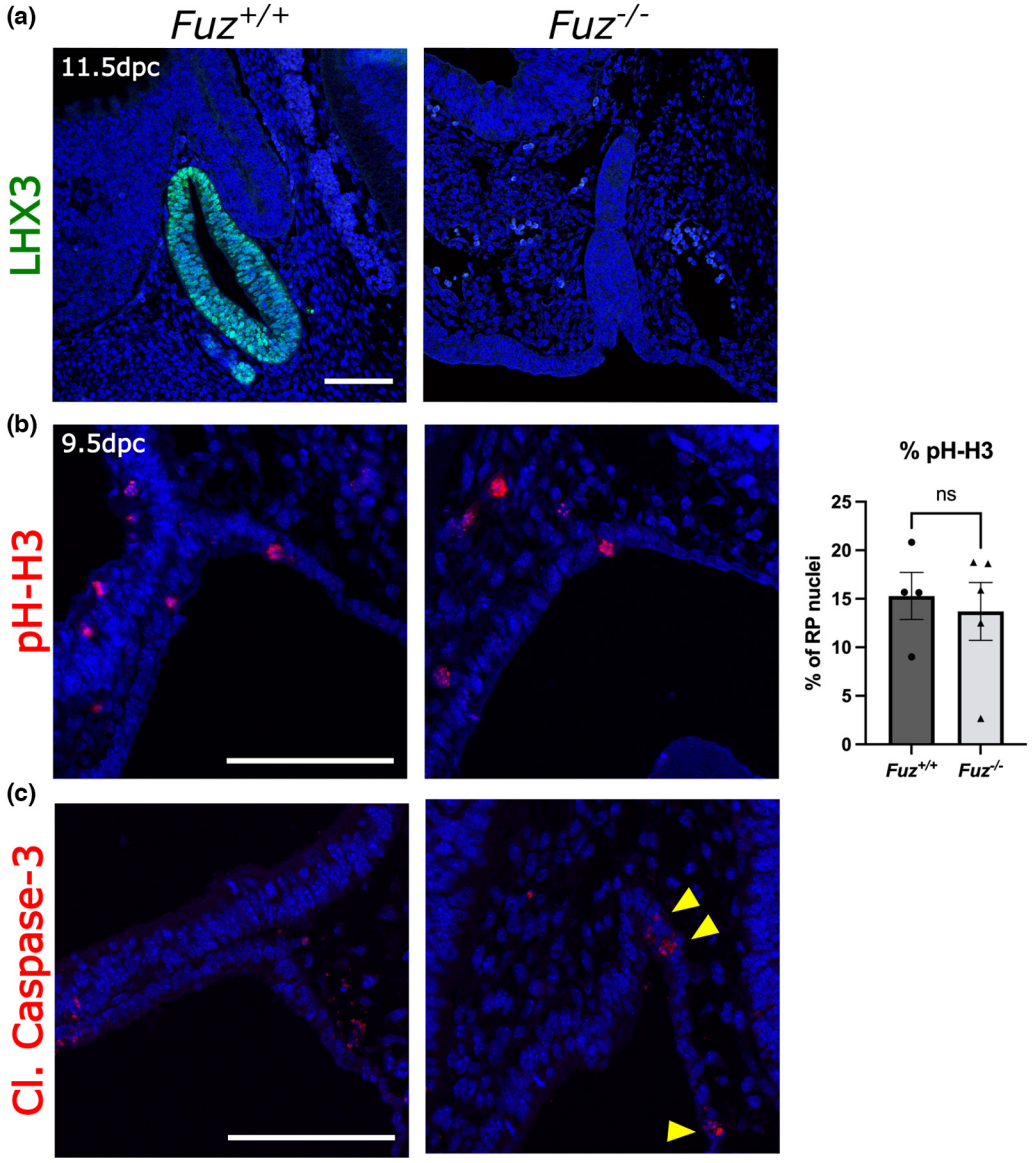
Rathke's pouch without *Fuz* loses pituitary identity and is lost via apoptosis. (a) Immunofluorescence staining of 11.5 dpc WT (*Fuz* ^*+/+*^) and *Fuz* ^*−/−*^ sagittal sections for pituitary transcription factor LHX3. There is no expression seen in *Fuz* ^*−/−*^. (b) Immunofluorescence staining of 9.5 dpc sections for pH‐H3 proliferation marker, and graph depicting counts of positive staining expressed as percent of nuclei forming RP, WT: 15.29%, *Fuz* ^*−/−*^: 13.69%, Student's t‐test, *p* > 0.05. WT *n* = 4 and *Fuz* ^*−/−*^ *n* = 5. (c) Immunofluorescence staining of 9.5 dpc for cleaved caspase‐3, active apoptosis marker, in WT where no expression is seen in RP, and *Fuz* ^*−/−*^ showing apoptotic cells throughout RP and PE (yellow arrowheads). DAPI nuclei counterstain shown in blue. All scale bars 100 μm.

To analyse the effects of the absence of FUZ protein on the cilia during pituitary development, we carried out immunofluorescence staining using antibodies against ciliary marker ARL13b. This revealed the presence of cilia along the VD (Figure 2a, dotted arrows) and within developing RP at 9.5 dpc in WT sections. However, in *Fuz* ^*−/−*^ mutants we observed almost a complete loss of cilia in the ventral diencephalon (Figure 2a, white arrow). Within RP, while we were able to identify cilia (Figure 3a, inset zoom) there was a reduction in the number (37.8% of RP cells were ARL13b + in WT vs. 16.9% of *Fuz* ^*−/−*^, *p* = 0.0019) (Figure 2a, unpaired *t*‐test, WT *n* = 4, *Fuz* ^*−/−*^ *n* = 5). We also observed cytoplasmic staining in the mutant pharyngeal endoderm (yellow arrowheads), which may indicate apoptotic cells. Staining at 11.5 dpc, confirmed the sustained presence of cilia in RP of both mutant and control (Figure S1D).

We next sought to identify if the defects in cilia in *Fuz* ^*−/−*^ mutants disrupt developmental signals necessary for RP development. In WT sections, *Shh* transcripts are expressed in the anterior hypothalamus (AH) and posterior hypothalamus (PH), as shown by RNAscope mRNA in situ hybridisation (Figure 2b, asterisks indicate the border of expression with VD/RP) but is not expressed in the VD. In addition, *Shh* is expressed in the pharyngeal endoderm (PE) caudal of RP. In contrast, in *Fuz* ^*−/−*^ sections, *Shh* transcripts are detected throughout the hypothalamus and diencephalon. Expression of *Shh* within the pharyngeal endoderm extends rostrally into RP (Figure 2b, yellow arrowheads). Furthermore, there is strong expression of *Shh* in cell clusters embedded in the mesenchyme, close to RP. Strikingly, there are also stripes of *Shh* transcripts in the midbrain (Figure 2b, yellow arrowheads), which were also observed in 11.5 dpc stained sections (Figure S1E). In control embryos at 9.5 dpc, expression of SHH target *Gli1* is detected in RP (Figure 2b, black arrows), midbrain and within mesenchymal cells. In *Fuz* ^*−/−*^ mutants, while some *Gli1* expression is seen within the RP region (Figure 2b, black arrows), this overlaps with *Shh* at 9.5 dpc. However, by 11 dpc, *Gli1* expression is reduced in the remnants of RP as well as the surrounding neural tissue and mesenchyme (Figure S1E).

Abnormal SHH signalling can perturb additional signals during pituitary development, specifically expression of *Fgf* and *Bmp* components, which will expand rostrally when *Shh* is absent (Carreno et al., 2017; Treier et al., 2001; Zhao et al., 2012). mRNA in situ hybridisation using probes against *Fgf8*, reveals the normal expression pattern in control samples at 9.5 dpc. *Fgf8* expression in the ventral diencephalon ends where the VD makes contact with RP (Figure 2c, black arrow). In *Fuz* ^*−/−*^ mutants the *Fgf8* expression domain is unaffected, but expression is weaker in VD, with apparent salt and pepper expression, and located primarily in basal regions of the tissue. There is ectopic expression of *Fgf8* in the oral epithelium at RP (Figure 2c, yellow arrowheads), and within the remnant BPM.

In the ventral diencephalon, *Bmp4* transcripts are expressed in a similar domain to *Fgf8* in control samples, with expression levels lower in the rostral domain of the neuroepithelium where it makes contact with the developing RP (Figure 2d, black arrows). In *Fuz* ^*−/−*^ mutant sections, mRNA in situ hybridisation with probes against *Bmp4* reveals dramatically reduced expression within the ventral diencephalon compared to controls, and ectopic expression of *Bmp4* along the oral epithelium, up to the rostral RP border (Figure 2d, yellow arrowheads).

### 2.3 Loss of FUZ results in loss of pituitary fate and tissue regression

To establish if the aberrant developmental signals influence RP specification, we carried out immunofluorescence staining for the essential pituitary transcription factor LHX3. At 11.5 dpc, LHX3 is expressed throughout RP in WT controls, whilst no expression is observed in the *Fuz* ^*−/−*^ mutant (Figure 3a). This confirms that RP is not specified correctly into an anterior pituitary cell fate.

To identify the cause of RP hypoplasia by 11.5 dpc, we carried out immunofluorescence staining using antibodies against pH‐H3, marking cells in G2/M. This identified proliferating cells in both the control and mutant RP at comparable levels (WT 15.29%, *Fuz* ^*−/−*^ 13.69%, *p* > 0.05, Figure 3b). Immunofluorescence staining at 11.5 dpc also revealed the presence of proliferating cells in the mutant (Figure S1F). To determine if the hypoplasia can be attributed to increased apoptosis, we carried out immunofluorescence staining using antibodies against cleaved caspase‐3 at 9.5 dpc. This revealed the presence of apoptotic cells within the *Fuz* ^*−/−*^ mutant RP, which are not observed in WT at this stage (arrowheads, Figure 3c). Similarly, we saw apoptotic cells within the RP at 11.5 dpc of *Fuz* ^*−/−*^ mutants, which are not observed in WT, however, both have cleaved caspase‐3‐positive cells in the OE underlying RP (Figure S1G).

Together these results confirm that within *Fuz* ^*−/−*^ mutants, RP initially forms, but is not specified correctly due to abnormal SHH, FGF and BMP signalling. This results in the lack of RP expansion and its regression, likely via apoptosis.

## 3 DISCUSSION

Clinical features of ciliopathies can include panhypopituitarism, isolated growth hormone deficiency, diabetes mellitus and hypogonadism (Focșa et al., 2021). We demonstrate that FUZ, a key component of the primary cilium, is required during pituitary development in mouse, in both the developing hypothalamus as well as Rathke's pouch. Genetic homozygous deletion of *Fuz* results in mutants where the invaginating RP becomes hypoplastic and its tissue is lost before 13.5 dpc through apoptosis. Mutant RP does not respond correctly to SHH signalling, which is abundantly expressed. Additionally, *Fgf8* and *Bmp4* expression in the ventral diencephalon are reduced at 9.5 dpc, a crucial time when RP required these signals. This could be due to elevated levels of *Shh* transcript, although the expression domain does not overlap with that of *Fgf8* and *Bmp4*. Additionally, this could be an independent manifestation on *Fgf8* and *Bmp4* signalling due to the lack of primary cilia in the ventral diencephalon. An ectopic expression of *Bmp4* in the oral epithelium is reminiscent of mutants lacking hypothalamic *Shh* expression (Carreno et al., 2017), suggesting that this tissue requires FUZ‐mediated response to SHH for correct patterning. The phenotype may be further complicated by abnormal positioning of the SHH‐secreting notochord, which can influence RP development (Khonsari et al., 2013). An abnormal notochord is a possibility in these mutants, which has not been formally investigated. The characterisation presented here suggests that the inability to respond to SHH underlies the defect, rather than the abundance of SHH. Collectively, aberrant signalling results in failure to express LHX3, indicative of failure to specify into an anterior pituitary cell fate. Loss of LHX3/LHX4 expression, result in regression of developing tissue (Sheng et al., 1997), also seen in mutants lacking SHH in the anterior hypothalamus (Carreno et al., 2017), where tissue reverts to an oral epithelial and pharyngeal endoderm fate. However, in *Fuz* ^*−/−*^ mutants we observed an increase in *Shh* expression throughout the diencephalon and hypothalamus, albeit with a reduction in *Gli1* effector expression, suggesting that the differences we see are due to poor signal transduction within the cell. While *Gli1* expression is not required for murine pituitary development, mutants for *Gli2* have been reported to have variable penetrance of complete pituitary loss at 12.5 dpc, and *Gli1/Gli2* double mutants, as well as *Gli2/Gli3* double mutants, were all reported to lack pituitary tissue (Park et al., 2000; Wang et al., 2010). Furthermore, *Gli2* mutants did not develop an infundibulum, and showed reduced expression of *Bmp4* and *Fgf8* in the ventral diencephalon at 9.5 dpc (Wang et al., 2010), similar to our observations in *Fuz* ^*−/−*^ mutants. We also observed the persistence of the buccopharyngeal membrane in *Fuz* ^*−/−*^ mutants at 9.5 dpc, tissue which has usually regressed before 9.0 dpc (Poelmann et al., 1985). A persistence of, or remnants of the membrane have also been reported in SHH null mutants and *Gli3* mutants (Tabler et al., 2014). This is consistent with our observations that SHH signalling is reduced in *Fuz* ^*−/−*^, despite an increase in *Shh* mRNA.

GLIs can also be processed into repressive forms, a process which is inhibited in *Fuz* mutants, which show a reduction in repressive GLI3R (Heydeck et al., 2009; Tabler et al., 2013), such that phenotypes observed may be due to a loss of said repression. Although in patients, loss of function GLI3 mutations are not associated with hypopituitarism or pituitary defects (Johnston et al., 2005). Rather mutations in *GLI3* which result in a functional, repressive protein (Johnston et al., 2005), are linked to Pallister Hall Syndrome (PHS) an autosomal dominant disorder characterised by postaxial polydactyly, imperforate anus, hypothalamic hamartoma and hypopituitarism (Clarren et al., 1980; Hall et al., 1980). A mouse model for PHS has been generated which exhibits most of the characteristic phenotypes (Böse et al., 2002); however, no pituitary defect has been identified in this mutant, suggesting that GLI3R activity during mouse pituitary development may be compensated, and that phenotypes we observe in *Fuz* ^*−/−*^ mice are more likely to be associated with an overall decrease in response to *Shh* than an increase in GLI3R‐mediated repression.

In the neural crest of *Fuz* ^*−/−*^ null mutants, FGF8 signalling was previously shown to be increased, and that some craniofacial phenotypes were rescued by heterozygous loss of FGF8 (Tabler et al., 2013). However, within the pituitary and ventral diencephalon of *Fuz* ^*−/−*^ mutants we observed a slight decrease in *Fgf8* expression, rather than an increase. This is likely due to a requirement for primary cilia in the ventral diencephalon, or due to an increase in *Shh* expression, which inhibits both *Fgf8* and *Bmp4* in these tissues (Carreno et al., 2017), or both. However, the *Shh* expression domain in mutants does not expand into the domain normally expressing *Fgf8* and *Bmp4*. Therefore, due to the different signalling manifestations between the neural crest and hypothalamic–pituitary axis, we do not expect that loss of FGF would rescue the pituitary phenotype of *Fuz* ^*−/−*^ mutants.

Aside from ciliogenesis, FUZ is also a Planar Cell Polarity (PCP) pathway effector, a pathway which we have previously examined in the pituitary in the context of receptor‐ligand FAT/DCHS protocadherins. Mutations in FAT/DCHS family result in pituitary phenotypes ranging from morphological defects of the anterior pituitary, interrupted pituitary stalk and ectopic posterior pituitary (Lodge et al., 2020 and see Voutetakis, 2021 for comprehensive review on pituitary stalk anomalies). However, these phenotypes do not overlap with what we see in *Fuz* ^*−/−*^ mutations, which appear consistent with defects in SHH signalling rather than PCP defects. Although FUZ is not directly involved in the SHH pathway, it is required for primary cilia formation, the location for signalling. We observed complete loss of cilia in the developing ventral diencephalon and hypothalamus, and reduced numbers in RP, likely affecting reception of SHH signals in both tissues and subsequently perturbing developmental signal expression from the hypothalamus, which can further influence RP development.

FUZ mutations in humans are linked to lethal neural tube defects, although a novel homozygous variant was found in surviving twins (Barrell et al., 2022). These monozygotic twins presented with craniosynostosis, however, also report precocious puberty, which may be due to a developmental hypothalamic–pituitary–gonadal axis defect. Further investigations in these patients, including pituitary imaging and a full endocrine work‐up could help identify the underlying cause.

FUZ forms part of the CPLANE complex with INTU and WDPCP and studies have suggested that both of these proteins might be able to compensate for loss of FUZ (Martin‐Salazar & Valverde, 2022). Mutations in WDPCP have been associated with Bardet‐Biedl syndrome (BBS), characterised by retinitis pigmentosa, obesity, kidney dysfunction polydactyly and hypogonadism (Kim et al., 2010), most commonly caused by pituitary defects (Mujahid et al., 2018). INTU and CPLANE1 mutations are also linked to Oral‐Facial‐Digital (OFD) syndrome, an association of abnormalities in the face, oral cavity and brachydactyly or polydactyly. There are reports of patients with OFD with pituitary abnormalities, including an absence of pituitary tissue (AL‐Gazali et al., 1999; Aljeaid et al., 2019; Buño et al., 2000; Shashi et al., 1995), although causative variants were not always identified in these patients, and may be due to mutations in other genes. These phenotypes also appear likely to be due to defects in SHH signalling, as midline defects are also described.

As such, there are multiple ciliopathies which have been associated with abnormal pituitary function, however, endocrine disturbances are rarely listed in ciliopathy phenotypes. The data presented implicate the primary cilium as an essential organelle for pituitary gland development, and support that endocrine disruption be considered and investigated in ciliopathy cases, as this can highly affect quality of life.

## 4 METHODS

### 4.1 Animals and tissue processing

Animal husbandry was carried out under compliance of the Animals (Scientific Procedures) Act 1986, Home Office licence and KCL ethical review approval. *Fuz* mutants (MGI:3531090) have previously been described (Gray et al., 2009). To generate embryos, timed matings between *Fuz* ^*+/−*^ × *Fuz* ^*+/−*^ were set up to produce *Fuz* ^*−/−*^ and WT littermates, where noon of the day of vaginal plug was designated as 0.5 dpc. Embryos were collected at 9.5 dpc, 11.5 dpc and 13.5 dpc and genotyped by a piece of tail tip.

Embryos were fixed in 10% neutral buffered formalin (Sigma, HT501128) overnight at room temperature, the next day tissue was washed and dehydrated through ethanol series, before being embedded in paraffin wax. Sagittal sections were cut at a thickness of 5 μm.

### 4.2 Histology

Paraffin sections were selected for appropriate axial level, to include Rathke's pouch, and stained with haematoxylin and eosin as previously detailed (Lodge et al., 2019).

### 4.3 mRNA in situ hybridisation

All mRNA in situ hybridisations were performed using the RNAscope 2.5 HD Reagent Kit‐RED (ACD Bio #322350) for single stains, or Duplex Reagent kit (ACD Bio #322430) for double *Shh* and *Gli1* staining. RNAscope in situ mRNA hybridisation was performed following manufacturer's protocol, except when using the Duplex kit the colours for Channel 1 and Channel 2 were switched by switching Amp 4 and Amp 8. Sections were mounted in VectaMount Permanent Mounting Medium (Vector Laboratories, H‐5000‐60). Probes used: *Fuz* (#451861), *Fgf8* (#313411)*, Bmp4* (#401301)*, Shh* (#314361) and *Gli1* (#311001‐C2).

### 4.4 Immunofluorescence staining

Sections were rehydrated through ethanol series, and antigen retrieval was performed in citrate buffer pH 6.0 using NXGEN decloaking machine at 110°C. Slides were washed in PBS‐Triton, blocked in blocking buffer (0.15% glycine, 2 mg/mL BSA, 0.1% Triton‐X in PBS) with 10% sheep serum and incubated in primary antibodies overnight, made up in blocking buffer with 1% sheep serum. The next day slides were washed and incubated in secondary antibodies made up in BB + 1% sheep serum for 1 h: Goat anti‐rabbit 594 (1:300 Abcam, ab150080) for pH‐H3, Cl. Caspase‐3 and LHX3 staining; Biotinylated goat anti‐rabbit (1:300, Abcam ab6720), for ARL13b staining. Slides were washed again, for ARL13b staining slides were incubated in Streptavidin‐555 (Life Technologies, S32355), made up in BB + 1% sheep serum for 1 h and then washed again. Finally, all slides were incubated in DAPI (1:5000, Abcam ab228549) in PBS for 10 min, then washed in PBS and coverslips were mounted with Vectashield Antifade Mounting Medium (Vector Laboratories, H1000‐10).

Primary antibodies used: pHH3 (rabbit, 1:300, Abcam ab5176), Cl. Caspase‐3 (rabbit, 1:300, Cell Signalling #9661S), LHX3 (rabbit, 1:300, Abcam ab14555) and ARL13b (rabbit, 1:300, Proteintech #17711‐1‐AP).

### 4.5 Imaging

Whole‐mount images were with a MZ10 F Stereomicroscope (Leica Microsystems), using a DFC3000 G camera (Leica Microsystems). Immunohistochemistry and RNAscope stained sections were taken with an Olympus BX34F microscope. Images of immunofluorescence staining were taken with a Zeiss LSM980 confocal microscope, using Zeiss Plan‐Apochromat 20×/0.8 dry objective, and a Zeiss Plan‐Apochromat 63×/1.40 Oil objective. Images were processed with Fiji (Schindelin et al., 2012).

Statistical analysis and graphs were produced using GraphPad Prism.

## AUTHOR CONTRIBUTIONS

EJL, CLA designed the study, interpreted data and wrote the manuscript. EJL performed experiments, collected and analysed the data. WBB, KJL provided crucial reagents and expertise.

## CONFLICT OF INTEREST STATEMENT

The authors have declared that no conflict of interest exists.

## Supporting information


Figure S1

## Data Availability

None.
