# Supplementary figures and images for: The Fuzzy planar cell polarity protein (FUZ), necessary for primary cilium formation, is essential for pituitary development
Source: J Anat. 2023 Oct 4;244(2):358–67. doi: 10.1111/joa.13961 (PMC10780146; doi:10.1111/joa.13961)

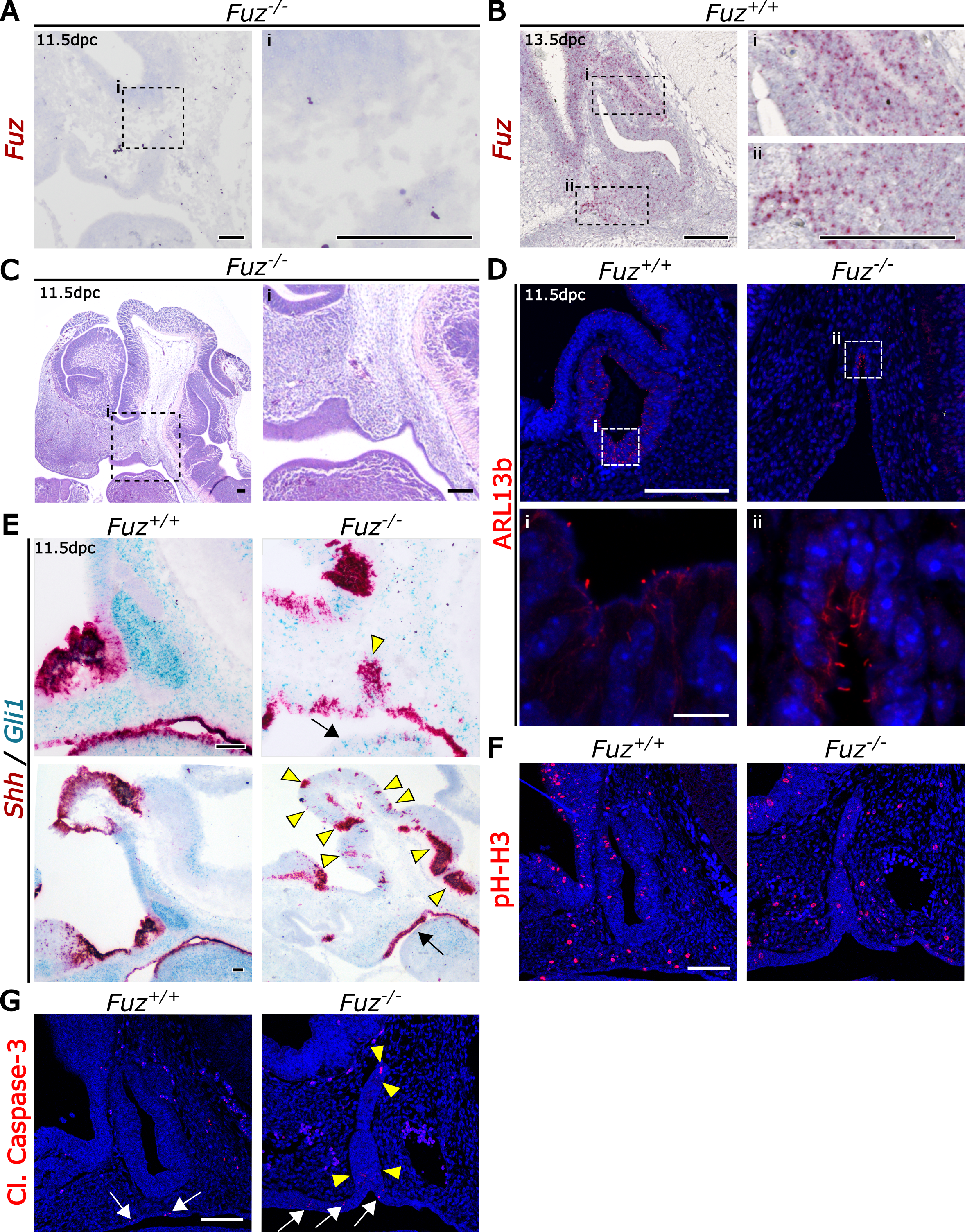

Supplement: Supplementary file 1 — Figure S1 [file JOA-244-358-s001.tif]
